# Biochemical characterization of the meiosis-essential yet evolutionarily divergent topoisomerase VIB-like protein MTOPVIB from *Arabidopsis thaliana*

**DOI:** 10.1093/nar/gkae181

**Published:** 2024-03-18

**Authors:** Hsin-Wen Chen, Hsin-Yi Yeh, Chih-Chiang Chang, Wei-Chen Kuo, Sheng-Wei Lin, Nathalie Vrielynck, Mathilde Grelon, Nei-Li Chan, Peter Chi

**Affiliations:** Institute of Biochemical Sciences, National Taiwan University, 10617 Taipei, Taiwan; Institute of Biochemical Sciences, National Taiwan University, 10617 Taipei, Taiwan; Institute of Biochemistry and Molecular Biology, College of Medicine, National Taiwan University, 100233 Taipei, Taiwan; Institute of Biochemistry and Molecular Biology, College of Medicine, National Taiwan University, 100233 Taipei, Taiwan; Institute of Biological Chemistry, Academia Sinica, 11529 Taipei, Taiwan; Université Paris-Saclay, INRAE, AgroParisTech, Institut Jean-Pierre Bourgin (IJPB), 78000,Versailles, France; Université Paris-Saclay, INRAE, AgroParisTech, Institut Jean-Pierre Bourgin (IJPB), 78000,Versailles, France; Institute of Biochemistry and Molecular Biology, College of Medicine, National Taiwan University, 100233 Taipei, Taiwan; Institute of Biochemical Sciences, National Taiwan University, 10617 Taipei, Taiwan; Institute of Biological Chemistry, Academia Sinica, 11529 Taipei, Taiwan

## Abstract

Formation of programmed DNA double-strand breaks is essential for initiating meiotic recombination. Genetic studies on *Arabidopsis thaliana* and *Mus musculus* have revealed that assembly of a type IIB topoisomerase VI (Topo VI)-like complex, composed of SPO11 and MTOPVIB, is a prerequisite for generating DNA breaks. However, it remains enigmatic if MTOPVIB resembles its Topo VI subunit B (VIB) ortholog in possessing robust ATPase activity, ability to undergo ATP-dependent dimerization, and activation of SPO11-mediated DNA cleavage. Here, we successfully prepared highly pure *A. thaliana* MTOPVIB and MTOPVIB-SPO11 complex. Contrary to expectations, our findings highlight that MTOPVIB differs from orthologous Topo VIB by lacking ATP-binding activity and independently forming dimers without ATP. Most significantly, our study reveals that while MTOPVIB lacks the capability to stimulate SPO11-mediated DNA cleavage, it functions as a *bona fide* DNA-binding protein and plays a substantial role in facilitating the dsDNA binding capacity of the MOTOVIB-SPO11 complex. Thus, we illustrate mechanistic divergence between the MTOPVIB-SPO11 complex and classical type IIB topoisomerases.

## Graphical Abstract

**Graphical Abstract:**
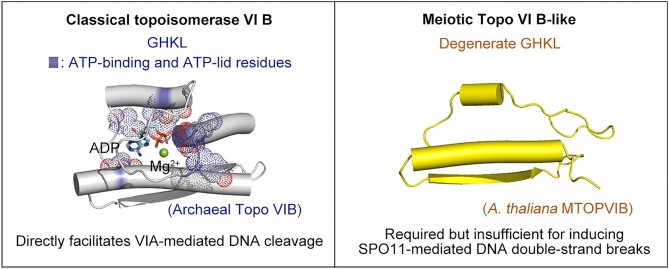

## Introduction

Meiosis plays an essential role in the sexual reproduction of most eukaryotic species and in the propagation of genetic diversity (1–3). During meiosis, recombination between homologous chromosomes is a vital event that facilitates the establishment and enhancement of homologous chromosome pairing, proper segregation of homologous chromosomes, and the generation of genetic diversity (4). Initiation of meiotic recombination relies on the formation of programmed double-strand breaks (DSBs), which are catalyzed by the evolutionarily conserved SPO11 (5–7). Mechanistically, it has been proposed that the conserved active-site tyrosine residue of SPO11 catalyzes a transesterification reaction by performing a nucleophilic attack on the DNA phosphodiester bond, resulting in the formation of a covalent phosphotyrosyl linkage between the protein and the 5′-end of the resulting DNA break (8–10). Then, the covalent SPO11-dsDNA oligo complex is released through endonuclease-mediated end resection. Subsequently, the exposed 3′ overhang of single-strand DNA (ssDNA) recruits the RAD51 and DMC1 recombinases to conduct homologous recombination and produce either reciprocal (crossovers) or non-reciprocal (non-crossover) exchange products (11–14).

SPO11 shares both sequence and functional similarities with the VIA subunit of archaeal Topo VI, a type IIB topoisomerase. Topo VI, which exists as a heterotetramer comprising two of each of the VIA and VIB subunits (15,16), resolves DNA topological problems arising from cellular DNA transactions by operating an elaborate catalytic cycle that starts from the production of a reversible DSB, followed by passing a second duplex DNA segment through this transient break, and finally re-joining the cleaved DNA to restore genome integrity (17,18). In the Topo VI complex, the Topo VIA subunit is responsible for DNA binding and cleavage. The VIA subunit consists of two domains, i.e. a winged-helix domain (WHD) for DNA binding and cleavage and a topoisomerase-primase domain (TOPRIM) that facilitates DNA cleavage by binding to Mg^2+^ (19–21). The VIB subunit contains the GHKL domain, which can bind and hydrolyze ATP and may function as an ATP-operated molecular clamp, along with the transducer domain that not only interacts with the VIA subunit but also bridges the communication between the GHKL domain and the VIA subunit (22,23). A cycle of ATP binding and hydrolysis by the VIB subunit is believed to induce conformational changes in the holoenzyme and stimulate the DNA cleavage activity of the VIA subunit (10,15,24–26). As such, the interplay between the Topo VIB subunit and ATP is crucial for Topo VI activity.

Like most Topo VIA orthologs, purified SPO11 protein appears to lack DNA cleavage activity (27–30), indicating that accessory factors are critical to the catalytic activity of SPO11. Consistent with this notion, genetic studies on *Arabidopsis thaliana* and *Mus musculus* have identified a distant homolog of the Archaea Topo VIB subunit (a meiotic Topo VIB-like protein, named MTOPVIB/TOPOVIBL), which interacts directly with SPO11 and is essential for generating meiotic DSBs *in vivo*. Notably, MTOPVIB orthologs from Embryophyta and Animalia share only limited structural and sequence homology with the archaeal Topo VIB subunit (31–33). The current consensus is that the catalytic activity accountable for DSB formation in eukaryotic meiosis has evolved from an ancestral topoisomerase function that is still retained in Archaea and certain eukaryotes (Topoisomerase VI) (34,35). Therefore, though MTOPVIB orthologs are considered to functionally approximate Topo VIB because they possess a degenerate version of the GHKL fold and harbor a transducer domain for interacting with SPO11 monomer (35,36), whether MTOPVIB possesses ATP binding and hydrolysis activities, as well as promotes SPO11’s DNA cleavage activity, has yet to be addressed experimentally.

In *A. thaliana*, both SPO11-1 and SPO11-2 are essential for the generation of meiotic DSBs. Mutating the catalytic tyrosine residue in either SPO11-1 or SPO11-2 cannot complement the meiotic defects resulting from SPO11 depletion, indicating that the enzymatic activities of both proteins are required for meiotic DSB formation (37,38). However, the interaction between SPO11-1 and SPO11-2 has only been detected in the presence of MTOPVIB in yeast three-hybrid analyses and bimolecular fluorescence complementation (BiFC) interaction assays *in planta*. Moreover, *mtopVIb* mutants exhibit a markedly defective meiotic DSB phenotype (31). These findings imply that MTOPVIB-mediated heterodimerization of SPO11-1 and SPO11-2 is a prerequisite for meiotic DSB formation. Nevertheless, the biochemical features of MTOPVIB and its functions in regulating SPO11-mediated DSBs have remained enigmatic due to a lack of highly purified protein for functional characterization. Here, we have successfully established an insect cell expression system and developed a purification and reconstitution procedure to obtain MTOPVIB, alone and in complex with SPO11-1 and SPO11-2, in a soluble and monodispersed form. Our biochemical and biophysical analyses reveal that MTOPVIB is a *bona fide* DNA-binding protein but, unlike archaeal Topo VIB, it lacks ATP-binding activity and does not activate SPO11-mediated DNA cleavage. We further show that MTOPVIB potentially acts as a *bona fide* and key DNA-binding subunit of the MTOPVIB-SPO11 complex. Collectively, our results highlight the functional divergence between MTOPVIB and its archaeal Topo VIB counterpart. These divergent biochemical features may be related to MTOPVIB-SPO11’s exaptation as the meiotic DSB generator.

## Materials and methods

### DNA substrates

The 2.9 kilobase (kb) supercoiled pBluescript II SK+ plasmid was purified from *Escherichia coli* using a plasmid midi kit (Qiagen). The linear form of pBluescript II SK+ was prepared by digesting the supercoiled DNA with *EcoRV* and purified using NucleoSpin^®^ Gel and PCR Clean-up kits (TaKaRa). The short DNA substrates for DNA-binding analysis—the 80-mer Oligo 1 (top-stranded ssDNA) (5′-TTATGTTCATTTTTTATATCCTTTACTTTATTTTCTCTGTTTATTCATTTACTTATTTTGTATTATCCTTATCTTATTTA-3′) and Oligo 1′s fully complementary Oligo 2 (bottom-stranded ssDNA) (5′-TAAATAAGATAAGGATAATACAAAATAAGTAAATGAATAAACAGAGAAAATAAAGTAAAGGATATAAAAAATGAACATAA-3′)—were synthesized and gel-purified by Genomics BioSci & Tech (Taiwan). To prepare 80 bp duplex DNA, a mixture of Oligo 1 and Oligo 2 was heated at 80°C for 3 min, followed by 65°C for 30 min, and then cooled down slowly to room temperature for DNA annealing. The DNA mixture was resolved in a 10% TBE polyacrylamide gel. After visualization under ultraviolet light, the annealed duplex DNA substrate was extracted by electro-elution and filter-dialyzed in a Centricon-10 concentrator (Millipore) at 4°C into TE buffer (10 mM Tris–HCl pH 8.0 and 0.5 mM EDTA). For surface plasmon resonance analysis, biotinylated 80 bp duplex DNA was annealed using 5′ biotin-labeled Oligo 1 (Genomics BioSci & Tech) and Oligo 2, as described above. The 80-bp DNA sequence we selected was designed to have a high AT content (82%), in agreement with the *in vivo* preference of *A. thaliana* SPO11-1 for AT-rich sequences (39), thereby ensuring that our binding assays are relevant to the biological interactions of SPO11-1.

For our DNA cleavage assay, in addition to pBluescript, we tested several *A. thaliana in vivo* recombination hotspot sequences (40) previously shown to correspond to preferential SPO11-1 cutting sites (39). The left and right segments of the 14a hotspot (known as 14a1 and 14a2, respectively), as well as the central region of the 130x hotspot, were amplified by primers (listed in Supplementary Table S1) and cloned into pCR™2.1-TOPO™ (3.9 kb) using a TOPO™ TA Cloning™ Kit (Invitrogen™). The 14a1, 14a2 and 130x sequences are 1656, 2132 and 680 bp in length, respectively, and their corresponding chromosomal positions are detailed in Supplementary Table S1. A 1349-bp region adjacent to the 14a hotspot, but that is deemed ‘cold’ for recombination (40), was included as a negative control (indicated as ‘coldspot’). All constructs were validated by sequencing.

### Protein expression plasmids

*A. thaliana MTOPVIB*, *SPO11-1* and *SPO11-2* full-length cDNAs (accession numbers Q5Q0E6, Q9M4A2 and OAP12700, respectively) were cloned separately into *XhoI* and *KpnI* sites of the pFastBac^TM^ Dual (Invitrogen) insect cell expression vector. For the purification strategy, six histidine-maltose-binding protein (His_6_-MBP), His_6_, and Flag tags were added separately to the N-terminus of MTOPVIB, SPO11-1 and SPO11-2. For tag removal, a PreScission protease cut site was inserted between the tag and protein sequences.

The codon-optimized cDNA of *Nanoarchaeum equitans* Topo VIB protein was cloned into *Nde1* and *Xho1* sites of the pET21b *Escherichia coli* expression vector. *N. equitans* Topo VI expression plasmid was constructed by inserting the codon-optimized cDNAs of *N. equitans* Topo VIA and Topo VIB separately into *BamH1–Not1* and *Nde1–Xho1* sites of pETDuet plasmid.

### Expression and purification of *A. thaliana* MTOPVIB and MTOPVIB-SPO11 complex

Individual baculoviruses harboring His_6_-MBP-*MTOPVIB*, His_6_-*SPO11-1* or Flag-*SPO11-2* were produced using a Bac-to-Bac Baculovirus Expression System (Invitrogen) according to the manufacturer's instructions. First, each pFastBac expression construct was transformed into DH10Bac^TM^ (Yeastern Biotech) *E. coli* cells to produce bacmid DNA. The bacmid DNA was extracted using a PureLink^TM^ HiPure Plasmid Midiprep Kit (Invitrogen), and the isolated bacmid DNAs were validated by polymerase chain reaction (PCR). For baculovirus generation, the verified bacmid DNAs were transfected into *Spodoptera frugiperda* (Sf9) insect cells, grown in suspension with serum-free ESF 921 medium containing 100 units/ml penicillin (Gibco) and 100 μg/ml streptomycin (Gibco), and then cultured in a non-humidified and non-CO_2_ incubator with 150 rpm shaking at 27°C. The resulting baculoviruses were harvested by centrifugation at 500 × *g* for 10 min, before filtering the media through a 0.2 μm filter and storing at 4°C in the dark.

To express proteins, High Five insect cells were infected by baculovirus with an appropriate volume of infection (VOI) (volume of media with virus (per ml)/volume of media with cells (per liter)). For His_6_-MBP-MTOPVIB expression, a VOI of 10 was used to infect High Five cells at a density of 2 × 10^6^ cells/ml. One liter of cultured cells was harvested after 36 hr incubation. Cell pellets were suspended with 200 ml A buffer (25 mM Tris–HCl pH 7.5, 0.5 mM EDTA, 10% glycerol, 0.02% Igepal, 1 mM β-mercaptoethanol, 10 mM ATP and 10 mM MgCl_2_) supplemented with 300 mM KCl, 2 mM Benzamidine, 0.2 mM PMSF and 2 μg/ml of a suite of protease inhibitors (Aprotinin, Chymostatin, Leupeptin and Pepstatin A), before being subjected to sonication. All of the protein purification steps were carried out at 4°C. After ultracentrifugation at 100 000 × *g* for 60 min, the clarified lysate was incubated with 5 ml Amylose resin (New England Biolabs) for 2.5 h. The slurry was extensively washed with 50 ml of A buffer supplemented with 150 mM KCl and eluted with 25 ml of A buffer supplemented with 150 mM KCl and 20 mM maltose. The eluted fractions were pooled together and diluted with an equal volume of A buffer and further applied to a 5 ml HiTrap™ Heparin HP column (GE Healthcare). The elution was fractionated with a 60 ml linear gradient of 75–630 mM KCl in A buffer. The MTOPVIB peak fractions were pooled and diluted with A buffer to make the salt concentration ∼150 mM KCl for application to a Mono Q 5/50 GL column (GE Healthcare). Following fractionation with a 45 ml gradient of 150–575 mM KCl in A buffer, the MTOPVIB-containing fractions were pooled and concentrated using a Centricon-30 concentrator (Millipore). The concentrated protein was further fractionated by size exclusion chromatography using a 24 ml Superdex 200 increase 10/300 GL column (GE Healthcare) in B buffer (A buffer without ATP and MgCl_2_) supplemented with 300 mM KCl. The soluble MTOPVIB fractions were pooled together and diluted with B buffer to 150 mM KCl. Then, PreScission protease (2 μg tagged MTOPVIB/μg protease) was added for overnight incubation at 4°C to remove the His_6_-MBP tag. The reaction mixture was fractionated through a 1 ml HiTrap^TM^ Heparin HP column (GE Healthcare) with a 15 ml gradient of 75–630 mM KCl in B buffer to separate MTOPVIB from the freed His_6_-MBP tag and PreScission protease. Finally, the non-tagged MTOPVIB-containing fractions were collected, concentrated using a Centricon-10 concentrator (Millipore), aliquoted, and stored at –80°C. The yield of MTOPVIB was almost 100 μg/l of infected insect cells.

*A. thaliana* MTOPVIB-SPO11 complex was expressed by infecting High Five cells with VOI = 5 of His_6_-MBP-*MTOPVIB* or VOI = 10 of His_6_-*SPO11-1* and Flag-*SPO11-2*. After 36 h incubation, the cell lysate from 1-l culture was purified through Amylose resin with C buffer (A buffer without β-mercaptoethanol) supplemented with 150 mM KCl. The eluent was further incubated overnight with ANTI-FLAG^®^ M2 Affinity Gel (Sigma-Aldrich). The resulting slurry was washed with 20 ml of buffer C containing 150 mM KCl and eluted with 10 ml of C buffer supplemented with 150 mM KCl and 200 μg/ml 3× Flag peptide (Sigma-Aldrich). Next, the elution fractions were applied to 5 ml HiTrap™ Heparin HP, Mono Q 5/50 GL, and Superdex 200 increase 10/300 GL columns, as described above for the purification of His_6_-MBP-MTOPVIB. The soluble MTOPVIB-SPO11 complex fractions from the size exclusion column were pooled together and concentrated using a Centricon-30 concentrator. The purified MTOPVIB-SPO11 complex was stored in small aliquots at -80°C. The yield of MTOPVIB-SPO11 complex was almost 20 μg/liter of infected insect cells.

### Expression and purification of *N. equitans* Topo VIB and Topo VI complex

*N. equitans* Topo VIB expression plasmid-harboring *E. coli* Rosetta (DE3) pLysS was grown at 37°C until the OD_600_ reached 0.4–0.5, at which time IPTG was added to a final concentration of 1 mM to induce protein expression at 37°C for 3 h. For protein purification, the cell pellet was resuspended in D buffer (20 mM Tris–HCl pH 8.0, 500 mM NaCl, 1 mM PMSF, 20 mM imidazole, 5 mM β-mercaptoethanol, and 10% glycerol) and then subjected to sonication. The crude cell lysate was clarified by centrifugation (three repeats) at 27 216 × *g* for 1 h at 4°C and applied to a Ni-NTA column. The column was washed to baseline, and the protein was eluted with D buffer containing 250 mM imidazole. The resulting protein sample was dialyzed against E buffer (20 mM Tris–HCl pH 7.5, 100 mM NaCl, 1 mM EDTA, 5 mM β-mercaptoethanol and 10% glycerol) and loaded into a HiPrep SP XL 16/10 column. The protein was eluted in a linear gradient over 10 column volumes with E buffer containing 1000 mM NaCl. The eluted fractions were pooled and further purified using a size-exclusion column (Hi-Load Superdex 200) in D buffer without imidazole and glycerol. Fractions containing the dimeric Topo VIB were collected and stored at –80°C. The yield of Topo VIB was almost 1000 μg/l of infected *E. coli* cells.

The *N. equitans* Topo VI complex was expressed and purified in a similar fashion to that described above for Topo VIB protein. In brief, the plasmid-harboring *E. coli* Rosetta (DE3) pLysS was grown at 37°C until the OD_600_ reached 0.6∼0.8, and protein expression was induced at 37°C for 4 h by adding IPTG to a final concentration of 1 mM. Then, the crude cell lysate was heated to 75°C for 1 h, centrifuged (three repeats) at 27 216 × *g* for 1 h, and purified via a Ni-NTA column with D buffer and a Hi-Load Superdex 200 column with E buffer containing 500 mM NaCl. Fractions containing the A_2_B_2_ tetrameric Topo VI were collected and stored at -80°C. The yield of Topo VI complex was almost 200 μg/l of infected *E. coli* cells.

### ATP-agarose pulldown assay

Adenosine 5′-triphosphate-Agarose (5′-ATP agarose) (Sigma-Aldrich) was used to analyze the ATP-binding ability of indicated proteins. *A. thaliana* MTOPVIB and *N. equitans* Topo VIB (2 μg each) were mixed with 10 μl 5′-ATP agarose in 20 μl of reaction buffer F (35 mM Tris–HCl pH 7.5, 1 mM DTT, 150 mM KCl) containing 200 μM MgCl_2_ and incubated at 23°C for 5 min with 1200 rpm shaking. The supernatant (S) was collected, and the resin was washed three times with 20 μl reaction buffer F containing 200 μM MgCl_2_. After the final washing step, the supernatant was collected as the wash (W), and the resin was treated with 20 μl 2× SDS sample dye (100 mM Tris–HCl pH 6.8, 4% SDS, 20% glycerol, 10% β-mercaptoethanol, 0.04% bromophenol blue) as the eluate (E). The S, W and E fractions were resolved in a 10% SDS polyacrylamide gel and stained with Coomassie blue solution.

### Surface plasmon resonance

The ATP- and DNA-binding abilities of MTOPVIB were determined using a Biacore T200 surface plasmon resonance instrument. For the ATP-binding analysis, *A. thaliana* MTOPVIB or *N. equitans* Topo VIB was immobilized on a CM5 sensor chip with an amine coupling kit. Then, 2-fold serially diluted ATP starting at a concentration of 100 μM was injected into the flow channels in reaction buffer F containing 200 μM MgCl_2_. For DNA-binding analysis, an 80 bp biotinylated dsDNA was immobilized on a streptavidin sensor chip. The two-fold serially diluted MTOPVIB starting at a concentration of 2 μM was injected into the flow channels in reaction buffer F supplemented with 0.05% Tween 20. Each condition was implemented at a flow rate of 30 μl/min for 2 min at 23°C, and the sensor surface was regenerated with 1 M NaCl and 50 mM NaOH before a new injection. The resulting signals were subtracted from the reference channel that had not been coated with ligands. Data were plotted in a resonance unit against the time sensorgram and the binding kinetics were established using BIAevaluation software (GE Healthcare). The ATP-binding affinity constant of *N. equitans* Topo VIB was determined according to the steady-state affinity model, and the DNA-binding affinity constant of *A. thaliana* MTOPVIB was determined by global fitting to the 1:1 binding model.

### Electrophoresis mobility shift assay (EMSA)

*A. thaliana* MTOPVIB, *A. thaliana* MTOPVIB-SPO11, *N. equitans* Topo VIB, or *N. equitans* Topo VI complex was incubated with 0.5 nM long DNA substrate (supercoiled or linear pBluescript) or 80 nM short DNA substrate (80 bp dsDNA, 80 mer top- or bottom-stranded ssDNA) in 10 μl of reaction buffer F containing 0.1 mg/ml bovine serum albumin (BSA) at 23°C for 30 min. The reaction mixtures were resolved in a 0.8% agarose gel in TBE buffer (89 mM Tris, 89 mM borate and 2 mM EDTA pH 8.0) at 4°C. To compare DNA-binding characteristics, the affinity tags of purified tagged MTOPVIB and MTOPVIB-SPO11 complex were removed by PreScission protease treatment immediately before undergoing DNA-binding assay. For the competitive DNA-binding analysis, 80 bp dsDNA and 80 mer top-stranded ssDNA were co-incubated with the indicated amount of MTOPVIB protein in a final reaction volume of 10 μl. The reaction mixtures were resolved in a 6% TBE polyacrylamide gel. All the DNA species were revealed by SYBR^®^ Gold (Invitrogen™) staining and detected using a Molecular imager^®^ Gel Doc™ XR^+^ station (Bio-Rad).

### DNA cleavage assay

*A. thaliana* MTOPVIB-SPO11 or *N. equitans* Topo VI complex was incubated with 0.5 nM supercoiled plasmid DNA substrates in 10 μl of reaction buffer F supplemented with 0.1 mg/ml BSA, 10 mM MgCl_2_, and 2 mM ATP at 75°C (Topo VI) or 23°C (non-tagged MTOPVIB-SPO11 complex) for 30 min. The DNA substrates included pBluescript, as well as pCR™2.1-TOPO™ harboring 14a1, 14a2, 130x hotspot, and the 14a-adjacent ‘cold’ region sequences. The non-tagged MTOPVIB-SPO11 complex was obtained from a PreScission protease cutting treatment immediately before DNA cleavage assay. Then, each reaction mixture was deproteinized by means of treatment with proteinase K (0.8 mg/ml) and SDS (0.1%) at 37°C for 15 min. To test the effect of various divalent cations on the DNA cleavage activity of the MTOPVIB-SPO11 complex, MgCl_2_ was replaced with 10 mM CaCl_2_, MnCl_2_, CoCl_2_ or ZnCl_2_. The reaction mixtures were resolved in a 0.8% agarose gel in TBE buffer and stained with SYBR^®^ Gold.

## Results

### Purification of *A. thaliana* MTOPVIB

**Figure 1.**
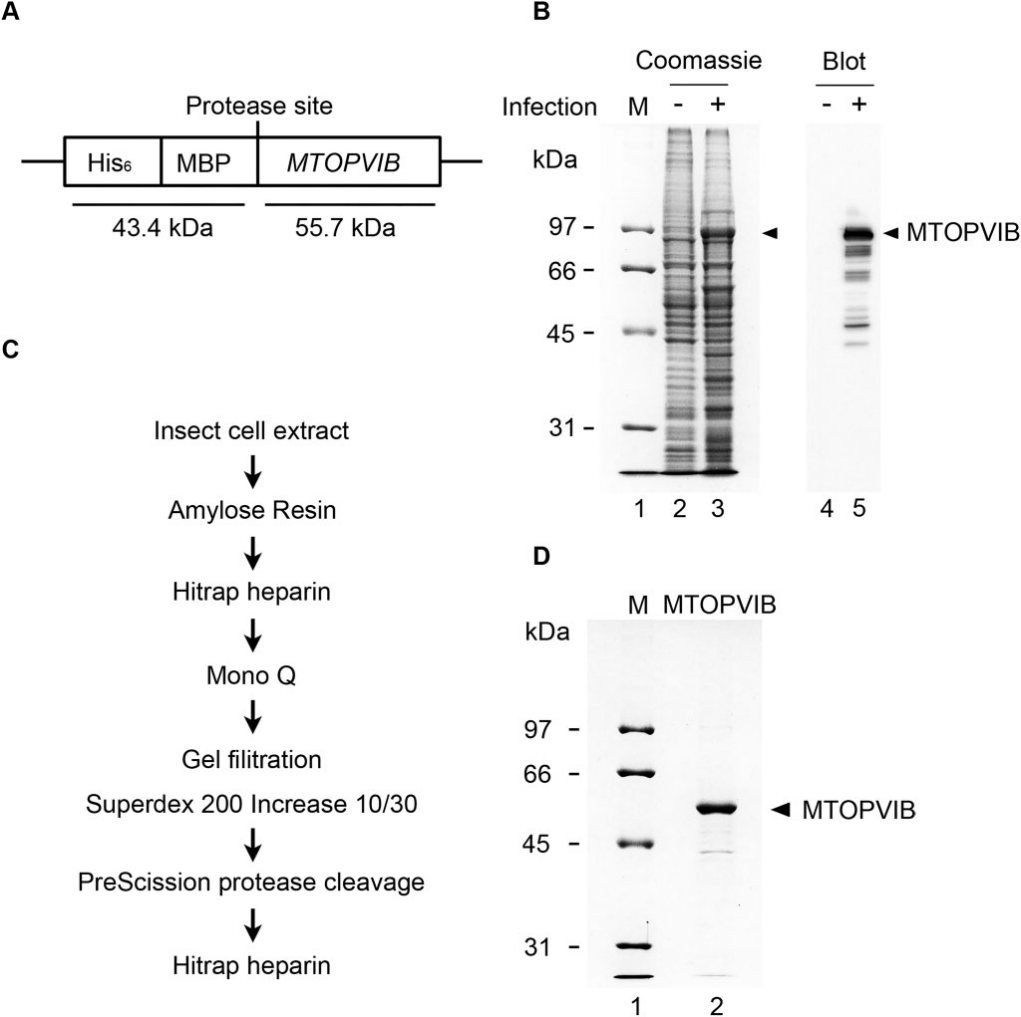
Expression and purification of *A. thaliana* MTOPVIB. (**A**) Diagram of the MTOPVIB expression construct. (**B**) Expression of MTOPVIB in High Five cells without (represented as ‘–’, lanes 2 and 4) or with (represented as ‘+’, lanes 3 and 5) baculovirus infection. Results were analyzed by 10% SDS-PAGE and stained with Coomassie Blue or by immunoblot analysis with α-His antibody (Sigma-Aldrich, H1029). (**C**) Purification scheme of non-tagged MTOPVIB. (**D**) Purified MTOPVIB (1 μg, lane 2), as resolved by 10% SDS-PAGE. Lanes 1 in (B) and (D) represent the protein markers.

To delineate the functions of MTOPVIB, we endeavored to prepare a soluble and monodispersed recombinant protein sample for biochemical characterizations. To this end, an expression plasmid harboring coding sequences for six histidines (His_6_), maltose-binding protein (MBP) with a C-terminal PreScission protease cutting site, and the *MTOPVIB* gene, in that order, was constructed for recombinant protein production in an insect cell expression system (Figure 1A). His_6_-MBP-MTOPVIB protein was successfully expressed in High Five insect cells, as verified by Western blotting (Figure 1B). Following a comprehensive purification procedure, including affinity, ion exchange, and size exclusion chromatography, as well as protease treatment (see Materials and Methods for details, Figure 1C), a non-tagged and monodispersed MTOPVIB recombinant protein was obtained. The purified MTOPVIB displayed near 95% purity and migrated in SDS-PAGE as a ∼55 kDa species (Figure 1D), consistent with its expected size. The identity of the purified MTOPVIB was further confirmed by liquid chromatography-tandem mass spectrometry (LC–MS/MS). The identified peptides cover 79 percent of the known MTOPVIB protein sequence (Supplementary Figure S1A). Several independent preparations of MTOPVIB gave comparable results and were used in all of the biochemical and biophysical experiments described below.

### *A. thaliana* MTOPVIB lacks ATP-binding activity

**Figure 2.**
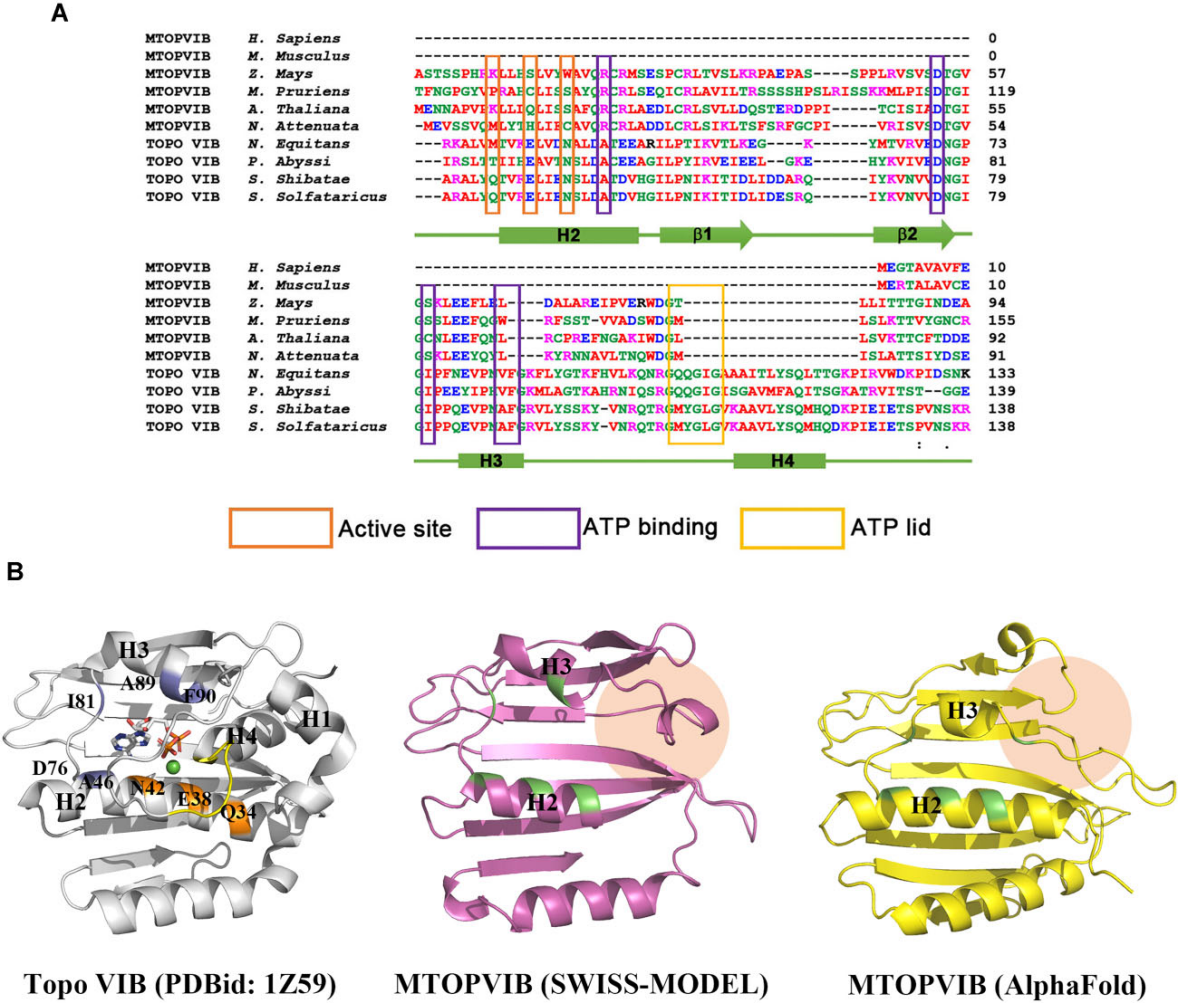
Structural modeling indicates a lack of ATP binding and hydrolysis activity for the GHKL domain of MTOPVIB. (**A**) Multiple sequence alignment of the GHKL domains of MTOPVIB and Topo VIB from representative species. MTOPVIBs from six different species (*Homo sapiens*, *Mus musculus*, *Zea mays*, *Mucuna pruriens*, *Arabidopsis thaliana*, *Nicotiana attenuata*) and Topo VIBs from four species (*Nanoarchaeum equitans*, *Pyrococcus abyssi*, *Saccharolobus shibatae*, *Saccharolobus solfataricus*) were included in the analysis. Residues involved in ATP hydrolysis, ATP binding, and those forming the ATPase lid region are indicated by orange, purple, and yellow boxes, respectively. (**B**) Structural modeling of the GHKL domains of MTOPVIB. The left panel displays the ribbon representation of *S. shibatae* Topo VIB (PDBid: 1Z59), in which residues and secondary structures involved in ATP binding and hydrolysis are labeled. The bound ADP molecule and Mg^2+^ ion are depicted as sticks and a green sphere, respectively. The middle and right panels show the structural models of the GHKL domain of *A. thaliana* MTOPVIB generated by SWISS-MODEL (middle, purple) and AlphaFold (right, yellow), respectively. Beige circles indicate the absence of helices H1, H4 and the H3–H4 loop, representing elements critical to ATP binding and hydrolysis, in MTOPVIB. Additionally, residues essential for ATP binding and hydrolysis, which are conserved in Topo VIB but altered in MTOPVIB, are marked in green in both the center and right panels.

**Figure 3.**
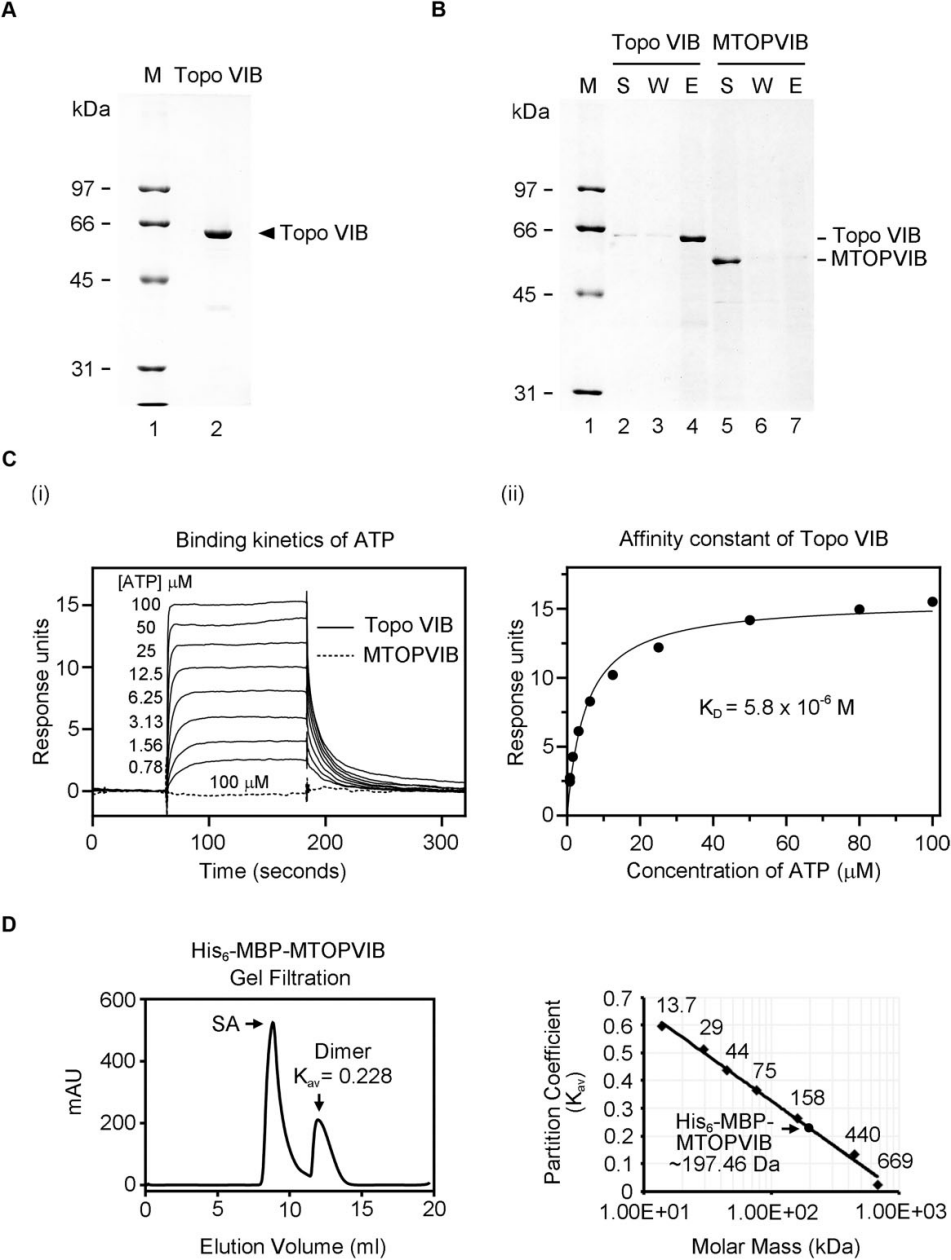
MTOPVIB, unlike archaeal Topo VIB, lacks ATP-binding activity and independently forming dimers without ATP. (**A**) Purified *N. equitans* Topo VIB (1 μg), as resolved by 10% SDS-PAGE. (**B**) The results of the ATP-agarose pulldown assay for Topo VIB and MTOPVIB were analyzed by 10% SDS-PAGE. ‘S’, ‘W’, and ‘E’ represent the supernatant, wash, and eluate fractions, respectively. (**C**) Surface plasmon resonance analysis of ATP-binding kinetics. (i) The ATP-binding kinetics of Topo VIB with gradually increasing ATP concentrations, as indicated, and MTOPVIB at a concentration of 100 μM. (ii) The equilibrium dissociation constant (*K*_D_) of ATP by Topo VIB according to the steady-state affinity model. (**D**) *A. thaliana* MTOPVIB is dimeric in solution. Purified MBP-tagged MTOPVIB was analyzed using a Superdex 200 10/300 GL column. The size standards of known molecular weight—aprotinin (13.7 kDa), carbonic anhydrase (29 kDa), ovalbumin (44 kDa), conalbumin (75 kDa), aldolase (158 kDa), ferritin (440 kDa) and thyroglobulin (669 kDa)—were analyzed according to the same protocol. Elution volume was determined by monitoring UV absorbance at 280 nm and the partition coefficient (*K*_av_) for each standard was calculated as (*K*_av_= (*V*_e_– *V*_0_)/(*V*_c_– *V*_0_)). The void volume (*V*_0_) of 8.358 ml was determined using Dextran Blue 2000 as the elution marker, while the column volume (*V*_c_) was ∼24 ml, as specified by the manufacturer for the prepacked columns. *V*_0_ = void volume, *V*_c_ = geometric column volume, *V*_e_ = elution volume. A plot of the partition coefficient (*K*_av_) versus molar mass using size standards was employed to calculate the apparent molecular weight of MBP-MTOPVIB. The two peaks in the gel filtration profile represent a soluble aggregation (SA) form and a dimeric form of recombinant MTOPVIB, respectively.

*A. thaliana* MTOPVIB is classically considered a homolog of the archaeal Topo VIB subunit. Intriguingly, despite their overall sequence similarity, specific residues involved in ATP binding and hydrolysis, which are strictly conserved in Topo VIB, appear to be either altered or missing altogether in MTOPVIB (Figure 2A). Consistent with sequence-based predictions, structural models of MTOPVIB generated independently using SWISS-MODEL and AlphaFold also indicated the absence from MTOPVIB of the lid region covering the ATP-binding pocket and an α-helix (H4) essential for mediating ATP binding and hydrolysis (Figure 2B). Collectively, these findings indicate that MTOPVIB may lack the ability to conduct ATP binding and hydrolysis. To explore that possibility experimentally, we examined the ATP-binding ability of MTOPVIB, using purified Topo VIB from the archaeon *N. equitans* as a positive control (Figure 3A). In our ATP-agarose pulldown assay, Topo VIB protein rather than MTOPVIB was pulled down by ATP-agarose. This result aligns with our sequence and structural modeling analyses, demonstrating that MTOPVIB lacks ATP-binding activity, whereas Topo VIB binds ATP tightly (Figure 3B).

In parallel with the ATP-agarose pulldown assay, we conducted surface plasmon resonance (SPR) to monitor the potential for transient protein-ATP interaction (Figure 3C). In this experiment, increasing concentrations of ATP were injected into the flow channels of a sensor chip harboring either immobilized Topo VIB or MTOPVIB. The ATP-binding kinetic curve showed dosage-dependent ATP-binding signals of archaeon Topo VIB as the concentration of ATP increased (Figure 3Ci). The equilibrium dissociation constant (*K*_D_: 5.8 × 10^−6^ M) for ATP binding by Topo VIB was determined according to the steady-state affinity model (Figure 3Cii). However, even at a concentration of 100 μM ATP, we did not detect any significant ATP-binding signal for MTOPVIB (Figure 3Ci). Thus, both our ATP-agarose pulldown and SPR analyses demonstrate that MTOPVIB harbors a degenerate GHKL domain that lacks ATP-binding activity.

### *A. thaliana* MTOPVIB is dimeric

Given that Topo VIB is known to undergo ATP-dependent dimerization and acts as an ATP-operated molecular clamp to facilitate topological changes of DNA, we sought to determine the oligomeric state of MTOPVIB in solution by means of size-exclusion chromatography. Using a Superdex 200 Increase 10/30 column calibrated by molecular weight standards, we established the elution profile of purified MBP-MTOPVIB as having an apparent size of ∼197.46 kDa, consistent with the calculated molecular weight of 198.2 kDa for the dimeric state (Figure 3D). Notably, formation of MTOPVIB dimer should be independent of MBP tagging due to the monomeric behavior of MBP in solution (41). Hence, our results suggest that, in contrast to classical Topo VIB, the dimerization process of *A. thaliana* MTOPVIB does not rely on ATP.

### MTOPVIB is a *bona fide* DNA-binding protein

**Figure 4.**
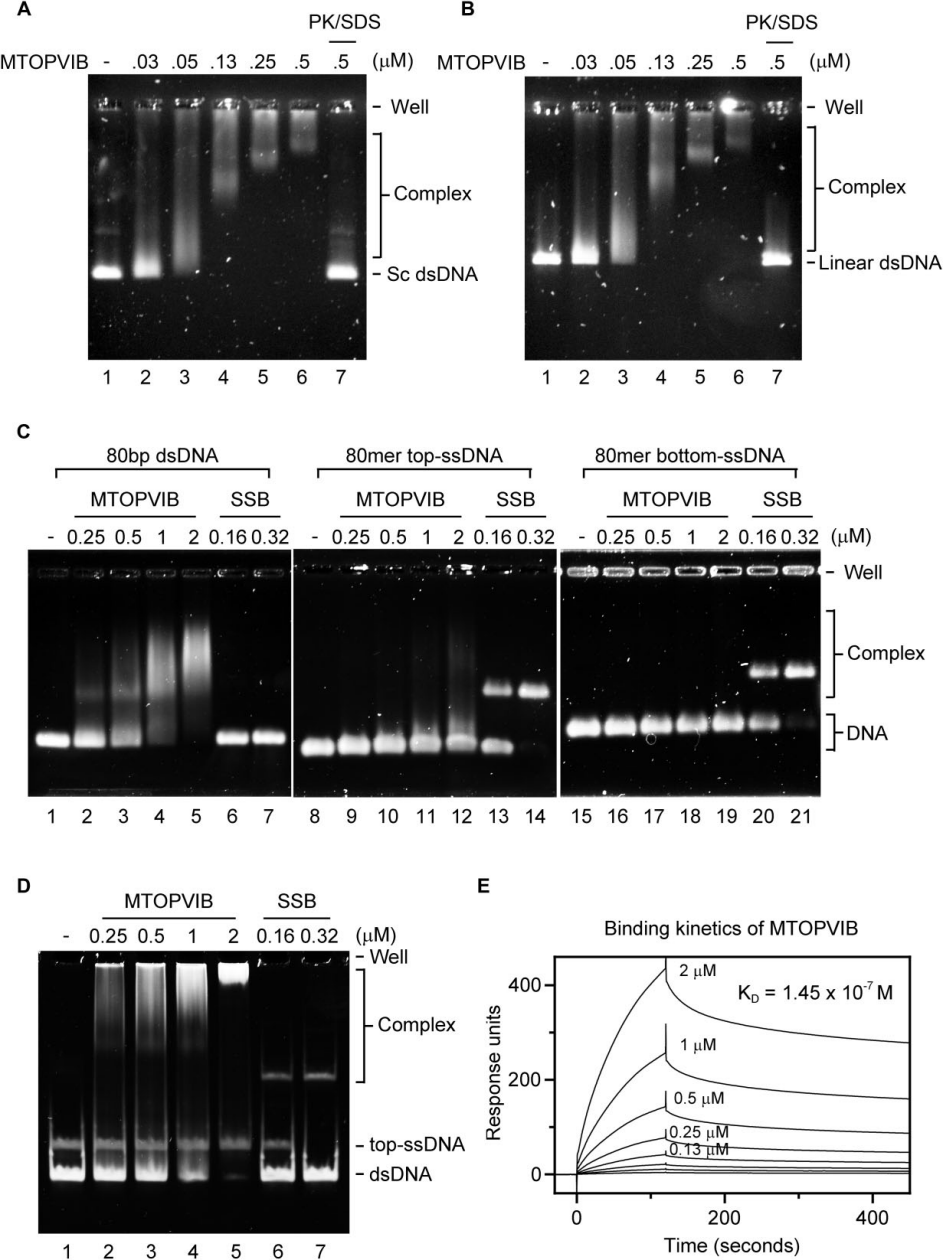
DNA-binding properties of MTOPVIB. (**A**) A negatively supercoiled form (Sc dsDNA) or (**B**) linearized form (Linear dsDNA) of double-stranded pBluescript plasmid DNA was used as the DNA substrate. To assess DNA-binding activity, the indicated concentration of MTOPVIB was incubated with the DNA substrate. In lane 7, the reaction mixture was treated with proteinase K (PK) and SDS to release DNA from the nucleoprotein complex, thereby confirming that the molecular weight shift in DNA species is due to direct MTOPVIB association. (**C**) Binding of MTOPVIB to the 80 bp dsDNA, as well as its constituent top- and bottom-ssDNAs, was tested. Single-stranded DNA binding protein (SSB) was included as the control. (**D**) The 80 bp dsDNA and top-ssDNA were co-incubated with the indicated amount of MTOPVIB to establish its DNA-binding preference. EMSA was performed in a 0.8% agarose-TBE gel for (A)–(C), and 6% TBE polyacrylamide gel for (D). (**E**) The binding kinetics of the 80 bp dsDNA with a gradually increased concentration of MTOPVIB (as shown) was measured by surface plasmon resonance (SPR). The equilibrium dissociation constant (*K*_D_) of MTOPVIB binding to DNA was determined by global fitting to a 1:1 binding model.

The DNA-binding activity of Topo VIB, mediated by the conserved KGRR loop and WKxY motif, has been well documented (42). In contrast, MTOPVIB possesses only the WKxY motif and lacks a recognizable KGRR sequence (31–33). To address if *A. thaliana* MTOPVIB possesses DNA-binding ability, we adopted electrophoresis mobility shift assay (EMSA) to monitor protein-DNA interactions. Our results show that MTOPVIB protein binds supercoiled or linear forms of plasmid DNA substrate in a dosage-dependent manner. Moreover, MTOPVIB protein displays no significant binding preference for either substrate type (Figure 4A and B). Next, we compared the binding affinity of MTOPVIB for ssDNA and dsDNA, using 80 mer ssDNAs (top- and bottom-strands of the duplex DNA) and duplex DNA as DNA substrates, respectively. Single-stranded DNA binding protein (SSB) was included as a control for ssDNA binding. Whereas dsDNA binding reached saturation at 2 μM MTOPVIB, we detected only minimal binding of ssDNA substrate under the same condition (Figure 4C). In a parallel analysis, we co-incubated ssDNA and dsDNA with MTOPVIB and then subjected them to mobility shift assay, which clearly revealed that MTOPVIB has a higher affinity for dsDNA (Figure 4D). Taken together, our results show that MTOPVIB possesses a significantly higher binding affinity for dsDNA. Finally, we determined by SPR the equilibrium dissociation constant for binding of MTOPVIB to dsDNA. To do so, an 80 bp biotinylated dsDNA was first immobilized on a streptavidin sensor chip. Then, purified MTOPVIB proteins were injected into the flow channels in a two-fold serial dilution, starting at a concentration of 2 μM. Our results indicate that the affinity constant for binding of dsDNA to MTOPVIB is ∼0.15 μM (Figure 4E).

### Purification of *A. thaliana* MTOPVIB-SPO11 complex

**Figure 5.**
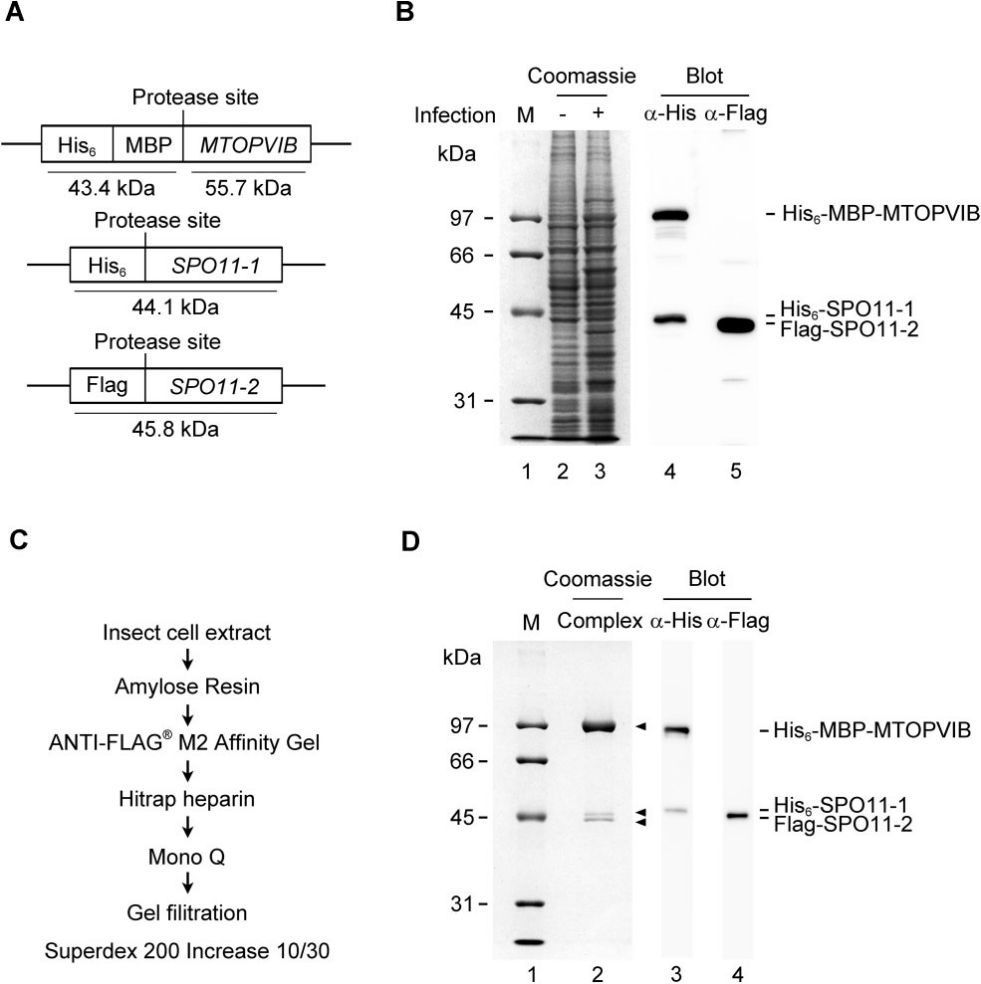
Expression and purification of *A. thaliana* MTOPVIB-SPO11 complex. (**A**) Diagrams of the MTOPVIB, SPO11-1, and SPO11-2 expression constructs. (**B**) Expression of the MTOPVIB•SPO11-1•SPO11-2 (MTOPVIB-SPO11) complex in High Five cells with or without baculovirus infection. (**C**) Purification scheme for the MTOPVIB-SPO11 complex. (**D**) Purified MTOPVIB-SPO11 complex was resolved by 10% SDS-PAGE. Results of immunoblot analysis in (B) and (D) were obtained using α-His (Sigma-Aldrich, H1029) or α-Flag-HRP (Sigma-Aldrich, A8592-2MG) antibody.

Previous genetic studies have documented that *A. thaliana* MTOPVIB, SPO11-1 and SPO11-2 are all required for the production of meiotic DSBs (31,37,38). Yeast three-hybrid and BiFC analyses have further indicated that the presence of MTOPVIB is necessary for formation of the MTOPVIB•SPO11-1•SPO11-2 (MTOPVIB-SPO11) complex (31). We deemed it important to determine if the MTOPVIB-SPO11 complex harbors DNA cleavage activity, so we purified the complex to high purity for functional study. First, we successfully coexpressed the three components in insect cells. Specifically, we attached a six-His tag and a Flag tag to the N-terminus of SPO11-1 and SPO11-2, respectively (Figure 5A). Western blotting verified that all three tagged proteins were expressed in the insect cells (Figure 5B). Then, the insect cell extract was subjected to a two-step affinity purification process (Amylose resin and anti-Flag affinity), before collecting the fractions of the MTOPVIB-SPO11 complex for further fractionation via Hitrap Heparin and Mono Q columns. A size-exclusion column was included in the final step to exclude the soluble aggregate and enhance MTOPVIB-SPO11 complex purity (Figure 5C). Co-purification of MTOPVIB, SPO11-1, and SPO11-2 confirmed the formation of MTOPVIB-SPO11 complex. The purified protein complex displayed nearly 90% purity, as revealed by a 10% SDS polyacrylamide gel (Figure 5D). Mass spectrometry and immunoblot analyses also confirmed the identity of all three components in the purified MTOVIB-SPO11 complex (Supplementary Figure S1B and Figure 5D). Notably, mass spectrometry results showed high coverage for MTOPVIB (72%), SPO11-1 (69%) and SPO11-2 (57%).

### The MTOPVIB-SPO11 complex exhibits a similar DNA-binding affinity to that of MTOPVIB

As described above, purified MTOPVIB lacks ATP-binding activity but displays significant binding to duplex DNA. Next, we examined by ATP-agarose pulldown assay if formation of the MTOVIB-SPO11 complex enables the ATP-binding activity of MTOPVIB. Whereas the Topo VI complex was pulled down in significant amounts, no MTOPVIB-SPO11 complex was captured by the ATP-agarose beads (Supplementary Figure S2A). To rule out the possibility that affinity tags may have affected protein binding, the tags appended to the MTOPVIB-SPO11 complex were removed by means of PreScission protease treatment. Again, no ATP-binding activity was detected for the tag-free MTOPVIB-SPO11 complex (Supplementary Figure S2B). Furthermore, we conducted a sensitive, radioisotope-based ATPase assay to examine the ATP hydrolysis activity of the MTOPVIB-SPO11 complex. However, unlike the Topo VI complex that hydrolyzed ATP to release γ-^32^Pi, the MTOPVIB-SPO11 complex exhibited no intrinsic ATPase activity (Supplementary Figure S2C).

**Figure 6.**
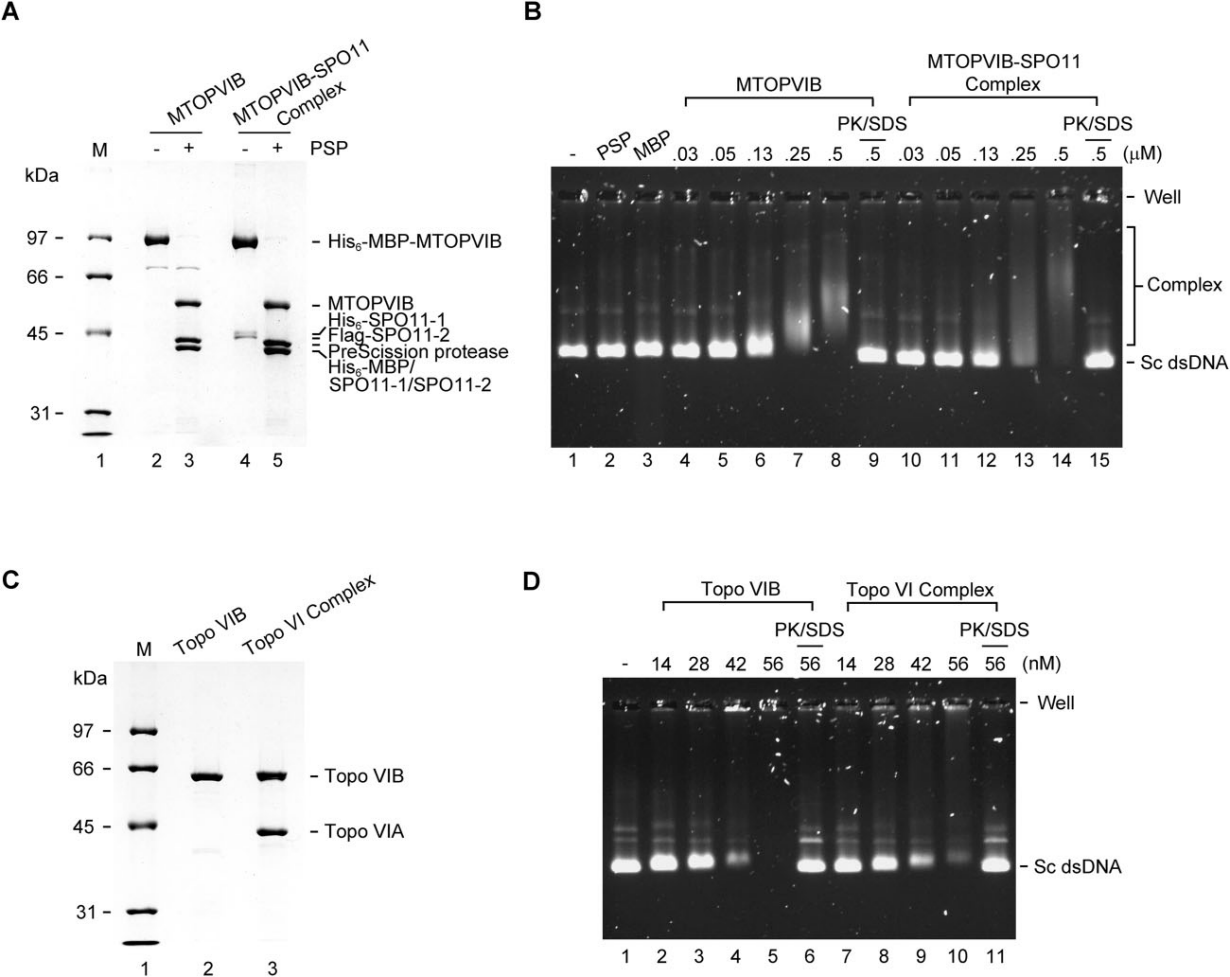
The B subunits of Topo VI and Topo VI-like MTOPVIB-SPO11 complexes contribute significantly to their dsDNA-binding activity. (A and C) Non-tagged MTOPVIB and MTOPVIB-SPO11 complex (**A**) or *N. equitans* Topo VIB and Topo VI complex (**C**) were resolved in 10% SDS-PAGE. (B and D) Supercoiled double-stranded DNA (Sc dsDNA) was incubated with the indicated amount of non-tagged MTOPVIB and MTOPVIB-SPO11 complex (**B**) or Topo VIB and Topo VI complex (**D**) to compare their DNA-binding affinities.

Next, we examined the DNA-binding characteristics of the MTOPVIB-SPO11 complex. Again, we removed the affinity tags of both purified tagged MTOPVIB and MTOPVIB-SPO11 complex by PreScission protease treatment for this set of experiments. The non-tagged MTOPVIB and MTOPVIB-SPO11 complexes were verified and quantified in a 10% SDS polyacrylamide gel (Figure 6A). Then the DNA-binding affinity of the tag-free MTOPVIB and MTOPVIB-SPO11 complex was measured by means of EMSA. Our results reveal that the MTOPVIB-SPO11 complex exhibits a similar DNA-binding affinity to that of MTOPVIB (Figure 6B). The DNA-binding activities of archaeal *N. equitans* Topo VIB and the Topo VI complex (Topo VIA•Topo VIB) were also examined for comparison (Figure 6C). We observed that whereas a 0.5 μM concentration of MTOPVIB-SPO11 was required to entirely shift the dsDNA substrate (Figure 6B), only 56 nM of the Topo VI complex was needed to achieve a similar effect (Figure 6D), indicating that Topo VI displays a stronger binding affinity for DNA than MTOPVIB-SPO11. Moreover, similar to MTOPVIB-SPO11, the Topo VI complex presented a comparable DNA-binding ability to that of Topo VIB (Figure 6D). These results indicate that the VIB and MTOPVIB subunits of the Topo VI and MTOPVIB-SPO11 complexes, respectively, contribute significantly to dsDNA binding. To further explore that possibility, it will be necessary to examine the DNA binding affinity of SPO11 and Topo VIA alone. Although it was not possible to generate *Arabidopsis* SPO11-1 and SPO11-2 to the required purity for such assays, we successfully isolated Topo VIA for a comparative DNA-binding study alongside Topo VIB and the Topo VI complex. The resulting EMSA data demonstrate that Topo VIB and Topo VI shifted the DNA substrate at a concentration of 56 nM, whereas Topo VIA failed to do so, even when we doubled the concentration (Supplementary Figure S3). This outcome points to a significant role for the B subunit in dsDNA binding within the Topo VI complex. Furthermore, our results indicate that the Topo VIB subunit and its meiotic homolog MTOPVIB play notable roles in the dsDNA binding activity of the Topo VI and MTOPVIB-SPO11 complexes.

### MTOPVIB-SPO11 complex is insufficient for dsDNA cleavage *in vitro*

**Figure 7.**
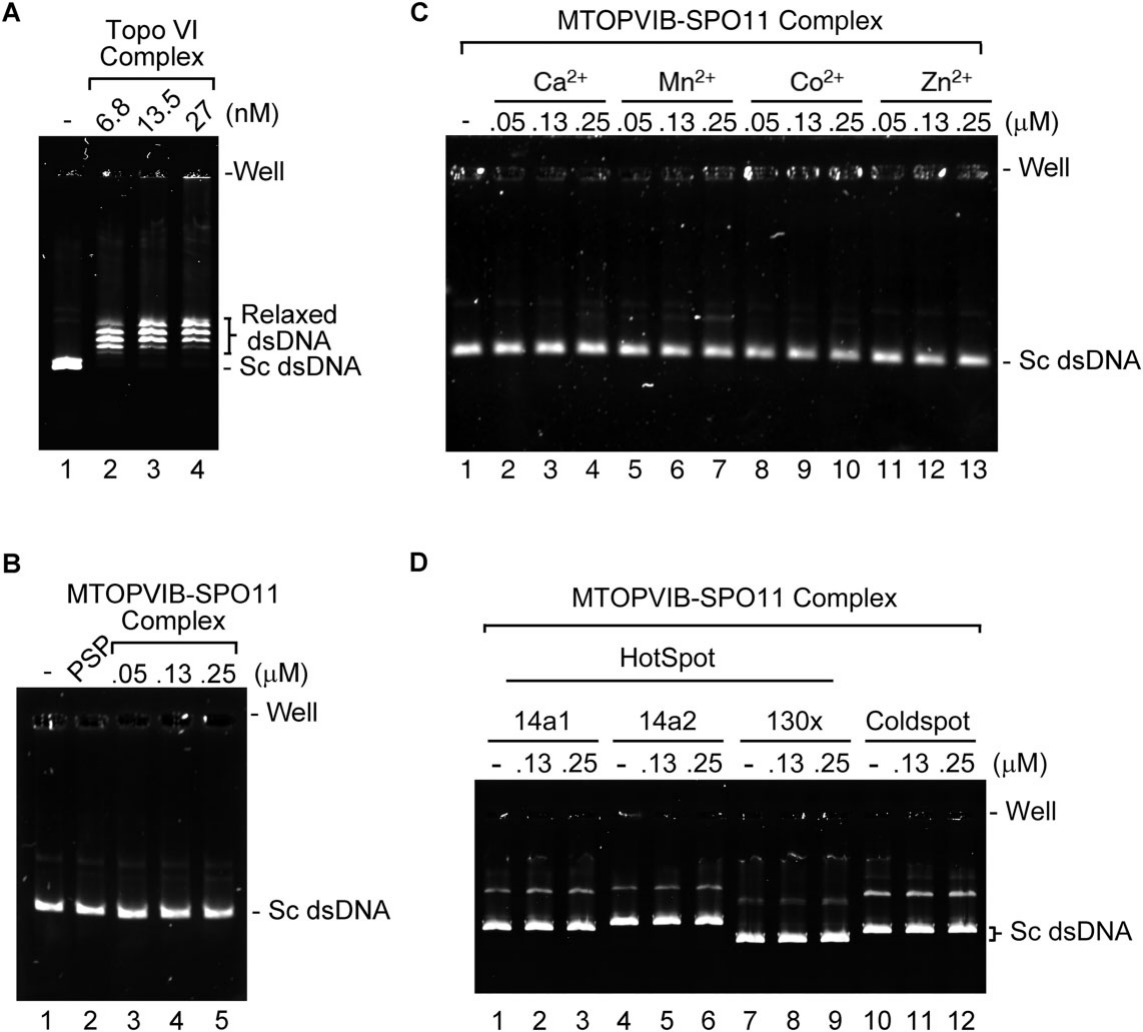
The *A. thaliana* MTOPVIB-SPO11 complex lacks DNA cleavage activity. The indicated concentration of Topo VI complex (**A**) and MTOPVIB-SPO11 complex (**B–D**) was incubated with negatively supercoiled plasmid DNA (Sc dsDNA) in 10 mM Mg^2+^ and 2 mM ATP. In (C), the Mg^2+^ was replaced with 10 mM Ca^2+^, Mn^2+^, Co^2+^ or Zn^2+^ to test the effect of various divalent cations on the DNA cleavage activity of the MTOPVIB-SPO11 complex. The DNA substrate used in (A–C) is pBluescript, whereas in (D) it is pCR™2.1-TOPO™ harboring the hotspot sequence 14a1, 14a2 or 130x. A genomic sequence ‘cold’ for recombination (‘coldspot’) was used as a negative control.

Finally, we investigated if MTOPVIB activates SPO11-mediated DNA cleavage activity. To this end, we analyzed the DNA cleavage activity of the purified MTOPVIB-SPO11 complex under various reaction conditions. The non-tagged MTOPVIB-SPO11 complex was incubated with negatively supercoiled DNA, pBluescript, in the presence of Mg^2+^ and ATP, followed by deproteinization of the reaction mixtures and analysis by agarose gel electrophoresis. We included *N. equitans* Topo VI as a positive control. Our results show that Topo VI exhibits DNA relaxation activity, whereas the MTOPVIB-SPO11 complex lacks DNA relaxation and DNA cleavage activities (Figure 7A and B). It has been reported previously that inclusion of divalent metal ions can stimulate the DNA relaxation activity of type II topoisomerases (43–45). However, we did not observe any significant cleavage of supercoiled DNA by the MTOPVIB-SPO11 complex in the presence of Ca^2+^, Mn^2+^, Co^2+^, or Zn^2+^ (Figure 7C). Additionally, we employed *A. thaliana in vivo* hotspot sequences as substrates to assess the DNA cleavage capability of the MTOPVIB-SPO11 complex. We inserted the established hotspot sequences, namely 14a1, 14a2, and 130x, into the pCR™2.1-TOPO™ vector to serve as substrates for the DNA cleavage assay. However, the MTOPVIB-SPO11 complex still did not appear to cleave these DNA substrates (Figure 7D). In conclusion, the MTOPVIB-SPO11 complex is insufficient alone to generate DSBs *in vitro*.

## Discussion

The essential role of Topo VIB-like proteins in meiotic DSB formation has been identified previously. Structural modeling has shown that the MTOPVIB-SPO11 complex likely adopts an overall architecture akin to that of Topo VI (34,35). However, a key question remained as to whether the meiotic Topo VIB-like proteins are functionally similar to Topo VIB in having ATP-binding activity and promoting SPO11-mediated DNA cleavage. Using highly purified *A. thaliana* MTOPVIB protein and MTOPVIB-SPO11 complex, we detected no ATP binding or DNA cleavage activities under various well-defined conditions. Interestingly, our results reveal that MTOPVIB, which shares with archaeal Topo VIB the highly conserved WKxY motif but lacks the KGRR loop, is a *bona fide* DNA-binding protein with an appreciable affinity for duplex DNA. Notably, MTOPVIB forms a complex with SPO11-1 and SPO11-2 and may substantially contribute to the DNA-binding affinity of that complex. Collectively, our findings provide the first biochemical characterization of the MTOPVIB/TOPOVIBL family and shed new light on the mechanistic divergence between classical type IIB Topo VI and the meiotic Topo VI-like core complexes.

### MTOPVIB harbors a degenerate GHKL fold without ATP-binding activity and is insufficient to promote SPO11-mediated DSBs

In archaeal Topo VI, the GHKL fold within the VIB subunit possesses ATPase activity and undergoes ATP-dependent dimerization. The transducer domain communicates structural signals from the ATP-binding site to the DNA breakage/reunion regions within the VIA subunit to coordinate DSB formation and DNA passage (24,26). Meiotic Topo VIB-like orthologs have been unveiled recently in many species (27,31,32). MTOPVIB/TOPOVIBL in plants and mice harbor a degenerate GHKL fold (with a poorly conserved ATP lid), as well as a transducer domain that interacts with SPO11 (31,32). The MTOPVIB/TOPOVIBL subunit of the budding yeast *Saccharomyces cerevisiae* comprises Rec104 as the GHKL fold and Rec102 as the transducer domain. However, Rec104 has no recognizable ATP binding and hydrolysis motifs (27). It is worth mentioning that a human TOPOVIBL ortholog has been identified by Robert *et al.* (32). A sequence alignment analysis performed by Robert *et al.* and us (Figure 2A) showed that the specific residues involved in ATP binding and hydrolysis are either altered or missing in human TOPOVIBL. In addition, using the AlphaFold structural model prediction, we also found that the ATP binding site does not exist in human TOPOVIBL when compared to *S. shibatae* Topo VIB (PDBid: 1Z59). Existing studies have extensively pointed out the degenerate nature of the GHKL fold, raising the question as to whether a lack of ATP-binding activity is a conserved feature of MTOPVIB/TOPOVIBL orthologs among the Embryophyta and Animalia. Here, our study documents that *A. thaliana* MTOPVIB lacks intrinsic ATP-binding activity. Most importantly, even the MTOPVIB-SPO11 complex does not display ATP-binding or ATP-hydrolysis activities. Although it cannot be ruled out that other accessory factors may induce MTOPVIB-mediated ATP binding, we speculate that the MTOPVIB-SPO11 complex could act differently to Topo VI in terms of DNA processing. Further biochemical studies on the MTOPVIB/TOPOVIBL subunit from other species are needed to verify this hypothesis.

### Ensemble of the MTOPVIB-SPO11 complex

The stoichiometry of Topo VI holoenzyme has been well characterized, revealing that the Topo VIA dimer interacts with each monomer of the B subunit to form an A_2_B_2_ heterotetramer (15,16,21,23,26). Unlike archaeal Topo VIA, purified *Caenorhabditis elegans* SPO11 exhibits a monomeric form in solution (29), and *A. thaliana* SPO11-1 and SPO11-2 dimerization occurs only in the presence of MTOPVIB (31). Interestingly, purified MTOPVIB behaved more like a dimer in our gel filtration analysis (Figure 3D), and affinity purification pulled down both SPO11-1 and SPO11-2 with MTOPVIB. Thus, our results suggest that dimeric MTOPVIB interacts with each SPO11 isoform and promotes heterodimerization of SPO11-1 and SPO11-2 in the context of the MTOPVIB-SPO11 complex. However, the precise stoichiometry of MTOPVIB and the MTOPVIB-SPO11 complex warrants further characterization. It is worth noting that the stoichiometry of the meiotic Topo VI-like complex may be divergent among species, as illustrated by the fact that purified *M. musculus* TOPOVIBL-SPO11 forms a 2:2 complex as a heterotetramer (32), whereas the *S. cerevisiae* Rec102-Rec104-Spo11-Ski8 complex displays a 1:1:1:1 stoichiometry (27).

### MTOPVIB possesses evolutionarily conserved DNA-binding activity

Structural and functional analyses of archaeal *Methanosarcina mazei* Topo VI revealed that the conserved WKxY motif and KGRR loop of Topo VIB are responsible for the efficient DNA-binding activity of Topo VI (42). The primary dsDNA-binding element, i.e. the WKxY motif in the transducer domain, is conserved among meiotic Topo VIB-like proteins (31,32,42), raising the question as to whether Topo VIB-like proteins possess intrinsic DNA-binding activity or if this activity is induced by the ensemble of the TopoVIA-B complex. Here, we have shown that both *N. equitans* Topo VIB and *A. thaliana* MTOPVIB exhibit intrinsic DNA-binding activity. Notably, Topo VIB is the primary DNA binder within Topo VI complex. Based on their DNA binding characteristics, the Topo VIB subunit, and by extension MTOPVIB, may be significantly involved in the dsDNA binding activity of Topo VI and MOTOVIB-SPO11 complexes. Identifying DNA binding-defective mutant variants of the MTOPVIB subunit will help delineate its functional role in regulating SPO11-mediated DNA cleavage. Our protein purification procedure presented in this work will allow us to isolate such mutants, enabling us to characterize their biochemical activities and structural features that are involved in generating DNA breaks.

### The SPO11-mediated DSB machinery

To date, biochemical evidence for the DNA cleavage activity of purified SPO11 recombinant proteins has not yet been documented. Accordingly, it has been proposed that certain SPO11-associated factors could somehow activate SPO11’s catalytic activity. Recently discovered Topo VIB-like proteins initially represented good candidates for such a role (31,32). However, here we have shown that the purified MTOPVIB-SPO11 complex is still insufficient alone to activate DNA breaks *in vitro*. This outcome indicates that additional proteins must be required for activating SPO11-mediated DNA cleavage. Consistent with this hypothesis, it has long been known that accessory proteins form various subcomplexes and are indispensable for DSB formation. Recent studies have indicated that axis elements and their DNA interactions mediate the formation of condensate-like clusters of SPO11 auxiliary factors that might trigger SPO11 activity. In *S. cerevisiae*, DNA-driven condensation of the Rec114-Mei4-Mer2 (RRM) complex has been proposed to act as a platform for recruiting the DSB core complex (Spo11-Rec102-Rec104-Ski8), on which Spo11 engages its DNA substrate and generates DNA breaks (27,46,47). In *Mus musculus*, Rec114 is a direct partner of TOPOVIBL, and the TOPOVIBL-Rec114 interaction ensures the efficiency and timing of meiotic DNA breaks (48,49). The DSB-promoting complex in the nematode *C. elegans* comprises DSB-1, DSB-2 (both predicted homologs of Rec114), and DSB-3 (a homolog of Mei4). This DSB-promoting complex controls meiotic DSB competence through the direct interaction of DSB-1 with SPO-11 (50–53). In *A. thaliana*, SPO11 accessory proteins also form aggregates on the axis and associate into various subcomplexes. A divergent RMM-like complex (including PHS1/AtREC114, PRD2/AtMEI4 and DFO) interacts with MTOPVIB and PRD1, establishing a connection between the DSB-catalytic MTOPVIB-SPO11 complex and its regulatory factors. Thus, the meiotic DSB machinery is anchored to the axis and DSB formation can be regulated (54,55). Accordingly, it is tempting to speculate that other components of the meiotic DSB machinery and their multiple interactions may contribute to regulating SPO11/MTOPVIB activity. *In vitro* reconstitution of the SPO11-mediated DSB machinery by including those purified components is the next step in elucidating the distinct mechanism of SPO11-mediated DSB formation.

## Supplementary Material

gkae181_Supplemental_File

## Data Availability

The relevant data are described in the Supplementary Information. Other additional data that support the findings of this study are available from the authors upon reasonable request.
