# Supplementary material for: Biochemical characterization of the meiosis-essential yet evolutionarily divergent topoisomerase VIB-like protein MTOPVIB from Arabidopsis thaliana
Source: Nucleic Acids Res. 2024 Mar 18;52(8):4541–55. doi: 10.1093/nar/gkae181 (PMC11077084; doi:10.1093/nar/gkae181)
Supplement: gkae181_Supplemental_File [file gkae181_supplemental_file.pdf]

Supplementary Information

Biochemical characterization of the meiosis-essential yet evolutionarily divergent topoisomerase VIB-like protein MTOPVIB from *Arabidopsis thaliana*

Hsin-Wen Chen[1,#], Hsin-Yi Yeh[1,#], Chih-Chiang Chang[2], Wei-Chen Kuo[2], Sheng-Wei Lin[3], Nathalie Vrielynck[4], Mathilde Grelon[4], Nei-Li Chan[2*], Peter Chi[1,3*]

[1] Institute of Biochemical Sciences, National Taiwan University, 10617 Taipei, Taiwan.

[2] Institute of Biochemistry and Molecular Biology, College of Medicine, National Taiwan University, 100233 Taipei, Taiwan.

[3] Institute of Biological Chemistry, Academia Sinica, 11529 Taipei, Taiwan.

[4] Université Paris-Saclay, INRAE, AgroParisTech, Institut Jean-Pierre Bourgin (IJPB), 78000, Versailles, France.

[#]These two authors contributed equally.

[*] To whom correspondence should be addressed:
Peter (Hung Yuan) Chi
Address:
Institute of Biochemical Sciences
College of Life Science
National Taiwan University
NO. 1, Sec. 4, Roosevelt Rd., Taipei, 10617 Taiwan
Email: peterhchi@ntu.edu.tw
Phone: 886-2-23665573
Fax: 886-2-23635038

Nei-Li Chan
Address:
Institute of Biochemistry and Molecular Biology
College of Medicine
National Taiwan University
No. 1, Jen Ai road section 1, Taipei, 100 Taiwan R.O.C.
Email: nlchan@ntu.edu.tw
Phone: 886-2-23562214
Fax: 886-2-23915295

This PDF file includes:
Supplementary Materials and Methods
Supplementary Figures S1 to S3
Supplementary Table S1

Supplementary Materials and Methods

Expression and purification of *N. equitans* Topo VIA

E. coli Rosetta (DE3) pLysS harboring the expression plasmid of *N. equitans* Topo VIA was grown at 37 °C until the OD_{600} reached 0.5~0.6. Protein expression was induced by 1 mM IPTG for 4 hr. Cells were collected by centrifugation and then re-suspended in D buffer (20 mM Tris-HCl pH 8.0, 500 mM NaCl, 1 mM PMSF, 20 mM imidazole, 1 mM β-mercaptoethanol, and 10% glycerol). After disrupting the cells by sonication, the crude cell lysate was centrifuged three times at 27,216 x *g* for 1 hr, and then the supernatant was applied to a Ni-NTA column and a size-exclusion column (Hi-Load Superdex 200) with D buffer without imidazole and glycerol. Fractions containing the dimeric Topo VIA were collected and stored at -80 °C.

ATPase assay

The ATPase activities of *A. thaliana* MTOPVIB-SPO11 and *N. equitans* Topo VI complexes were examined in the presence of plasmid DNA. MTOPVIB-SPO11 or Topo VI complex (0.5 μM) was incubated with 0.25 μM supercoiled pBluescript in 10 μl of reaction buffer (35 mM Tris-HCl pH 7.5, 1 mM DTT, 150 mM KCl, 10 mM $MgCl_2$, 100 ng/μl BSA, 5 μM ATP, and 5 μCi of [γ-^{32}P]ATP) at 23 °C (non-tagged MTOPVIB-SPO11 complex) or 75 °C (Topo VI) for the indicated times (10, 20, 40 mins). The affinity tags of purified tagged MTOPVIB-SPO11 complex were removed by PreScission protease treatment immediately before undergoing ATPase assay. Aliquots (2 μl) were taken at the indicated times and mixed with an equal volume of 500 mM EDTA. ATP hydrolysis was determined by thin-layer chromatography on a PEI-TLC plate (MACHEREY-NAGEL) using 0.5 M LiCl and 1 M formic acid as the developing buffer. The chromatography sheet was air-dried and subjected to phosphorimaging analysis.

Supplementary Figure S1

A

MTOPVIB

```
  1 MENNAPVPKL LLQLISSAFQ RCRLAEDLCR LSVLLDQSTE RDPPITCISI
 51 ADTGIGCNLE EFQNLRCPRE FNGAKIWDGL LSVKTTCFTD DEVYYYHINL
101 DEYIANKRLK RQPSQAKNGA KFSGTEVSLS VFGSMDVLVA PIIGFFQKII
151 VLQILNVTLD LMVKQGTSPG NQTQYVFAVN ADKTPCFTAS NLERLKSGLE
201 DYVLRHANCL DTMCDYCFSD REHLKVGSGT VCQEDKHKRV GGTMEVVIVI
251 SDLLESTQHC SRSCNGKTEV LYFDNFLPSP VPHLALSALK KIDWKKYGLI
301 LANVNDQDGH VFLEWDNFPS YVQIQIALHW YHNQYPTRQK NGPGISLLKK
351 GIKNALDNLK AKHEGFLLSS HSRKICSYVP DLARSIAGLI FSSTDLDFQG
401 GDCLSVLGFQ TQEVERDTVE NYIQRKIVTV IGMNERKPQK DQEAAPFLFF
451 DGESETSFFE DEEVEDRENS THEYDATATK AYTQFKCLAT MLQE
```

B

MTOPVIB

```
  1 MENNAPVPKL LLQLISSAFQ RCRLAEDLCR LSVLLDQSTE RDPPITCISI
 51 ADTGIGCNLE EFQNLRCPRE FNGAKIWDGL LSVKTTCFTD DEVYYYHINL
101 DEYIANKRLK RQPSQAKNGA KFSGTEVSLS VFGSMDVLVA PIIGFFQKII
151 VLQILNVTLD LMVKQGTSPG NQTQYVFAVN ADKTPCFTAS NLERLKSGLE
201 DYVLRHANCL DTMCDYCFSD REHLKVGSGT VCQEDKHKRV GGTMEVVIVI
251 SDLLESTQHC SRSCNGKTEV LYFDNFLPSP VPHLALSALK KIDWKKYGLI
301 LANVNDQDGH VFLEWDNFPS YVQIQIALHW YHNQYPTRQK NGPGISLLKK
351 GIKNALDNLK AKHEGFLLSS HSRKICSYVP DLARSIAGLI FSSTDLDFQG
401 GDCLSVLGFQ TQEVERDTVE NYIQRKIVTV IGMNERKPQK DQEAAPFLFF
451 DGESETSFFE DEEVEDRENS THEYDATATK AYTQFKCLAT MLQE
```

SPO11-1

```
  1 MEGKFAISES TNLLQRIKDF TQSVVVDLAE GRSPKISINQ FRNYCMNPEA
 51 DCLCSSDKPK GQEIFTLKKE PQTYRIDMLL RVLLIVQQLL QENRHASKRD
101 IYYMHPSAFK AQSIVDRAIG DICILFQCSR YNLNVVSVGN GLVMGWLKFR
151 EAGRKFDCLN SLNTAYPVPV LVEEVEDIVS LAEYILVVEK ETVFQRLAND
201 MFCKTNRCIV ITGRGYPDVS TRRFLRLLME KLHLPVHCLV DCDPYGFEIL
251 ATYRFGSMQM AYDIESLRAP DMKWLGAFPS DSEVYSVPKQ CLLPLTEEDK
301 KRTEAMLLRC YLKREMPQWR LELETMLKRG VKFEIEALSV HSLSFLSEVY
351 IPSKIRREVS SP
```

SPO11-2

```
  1 MEESSGLSSM KFFSDQHLSY ADILLPHEAR ARIEVSVLNL LRILNSPDPA
 51 ISDLSLINRK RSNSCINKGI LTDVSYIFLS TSFTKSSLTN AKTAKAFVRV
101 WKVMEICFQI LLQEKRVTQR ELFYKLLCDS PDYFSSQIEV NRSVQDVVAL
151 LRCSRYSLGI MASSRGLVAG RLFLQEPGKE AVDCSACGSS GFAITGDLNL
201 LDNTIMRTDA RYIIIVEKHA IFHRLVEDRV FNHIPCVFIT AKGYPDIATR
251 FFLHRMSTTF PDLPILVLVD WNPAGLAILC TFKFGSIGMG LEAYRYACNV
301 KWIGLRGDDL NLIPEESLVP LKPKDSQIAK SLLSSKILQE NYIEELSLMV
351 QTGKRAEIEA LYCHGYNYLG KYIATKIVQG KYI
```

Supplementary Figure S1. Mass spectrometry analysis of *A. thaliana* MTOPVIB and the MTOPVIB-SPO11 complex. Identified fragments of **(A)** purified MTOPVIB protein and **(B)** the three components of the MTOPVIB-SPO11 complex have been underlined.

Supplementary Figure S2

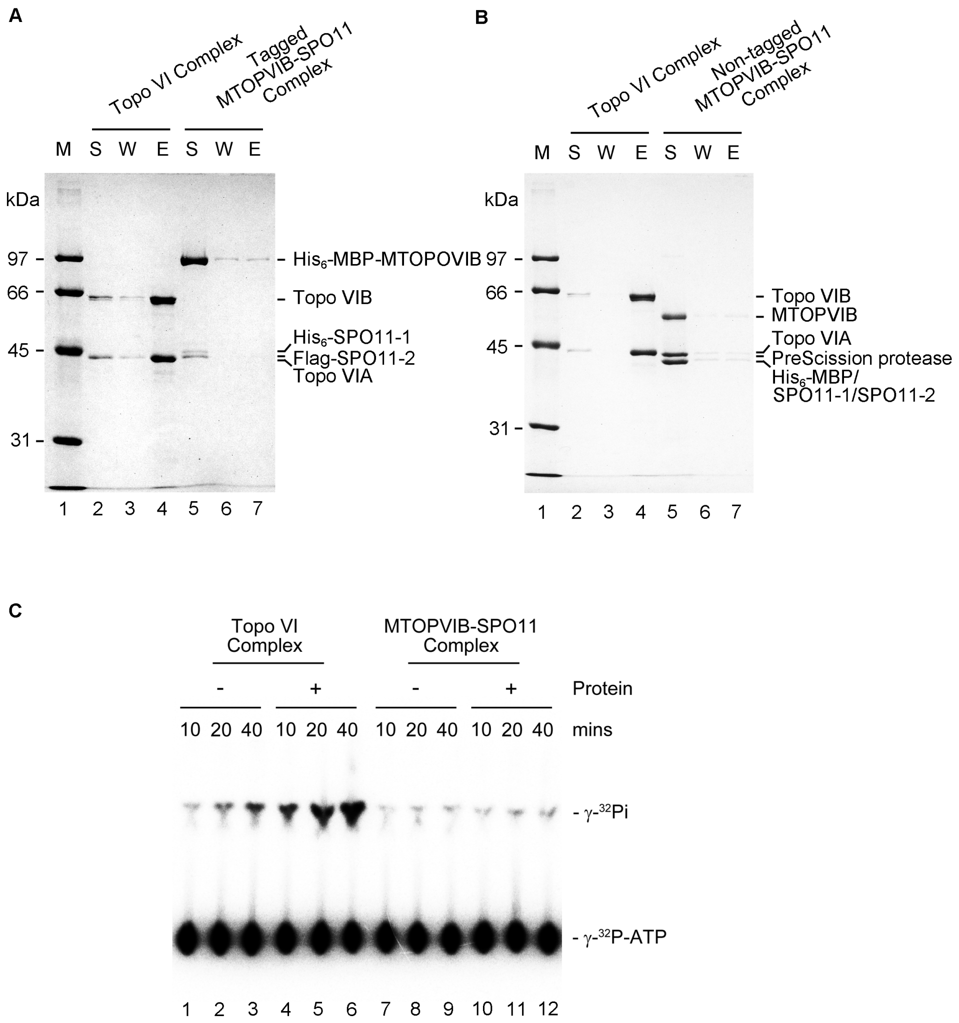

Supplementary Figure S2. The *A. thaliana* MTOPVIB-SPO11 complex lacks ATP-binding and hydrolysis activities. (A and B) ATP-agarose pulldown assay for the *A. thaliana* MTOPVIB-SPO11 complex. Purified tagged (A) and non-tagged MTOPVIB-SPO11 complex (B) were used for ATP-agarose pulldown analysis. The *N. equitans* Topo VI complex was included as the positive control. All analytes were resolved by 10% SDS-PAGE. "S", "W", and "E" represent the supernatant, wash, and eluate, respectively. **(C)** ATPase assay for the *A. thaliana* MTOPVIB-SPO11 complex. In brief, the Topo VI or MTOPVIB-SPO11 complex was incubated with supercoiled pBluescript in the presence of [γ-^{32}P]ATP for the indicated times (10, 20, 40 mins). Reactions were stopped and ATP hydrolysis activity was then determined by thin-layer chromatography. In (B) and (C), the affinity tags of purified tagged MTOPVIB-SPO11 complex were removed by PreScission protease treatment immediately before analyses.

Supplementary Figure S3

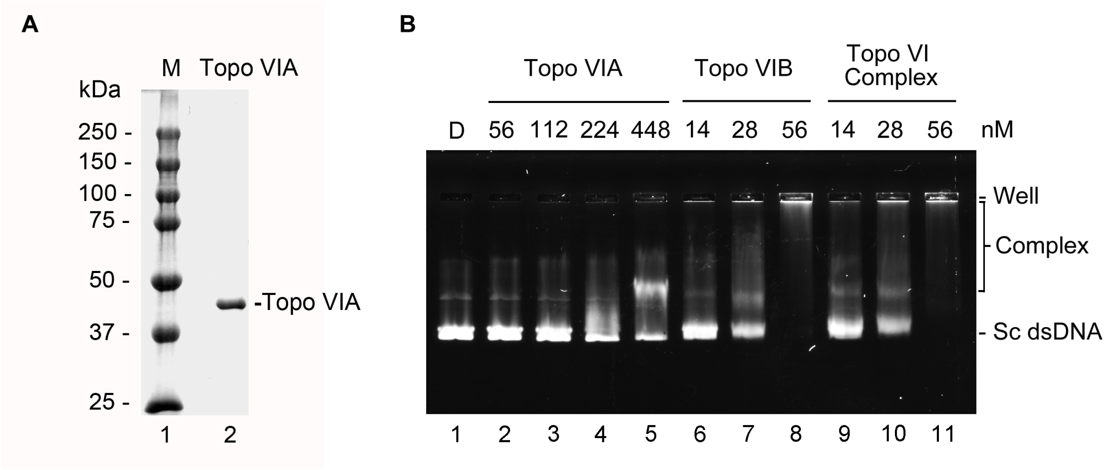

Supplementary Figure S3. The B subunit of the Topo VI complex contributes significantly to dsDNA-binding activity. (A) Purified *N. equitans* Topo VIA was resolved by 10% SDS-PAGE. **(B)** The indicated concentrations of *N. equitans* Topo VIA, Topo VIB, and Topo VI complex were incubated with supercoiled pBluescipt dsDNA (Sc dsDNA) at 23 °C for 30 min. The reaction mixtures were resolved in a 0.8% agarose gel in TBE buffer and stained with SYBR® Gold.

Supplementary Table S1. Amplification primers for the *A. thaliana in vivo* recombination hotspot sequences and their corresponding chromosomal positions.

Region amplified	Forward primer sequence (5’-3’)	Reverse primer sequence (5’-3’)	Position on chromosome 4
Hotspot 14a1	ATCTTATAAACGTTATTGTCA	TGTAATTCAACTGCGCTTGTA	16,833,394 – 16,835,049
Hotspot 14a2	TAAAAGGTTATCTCAAGAAG	TGCCCAATTTACAATCTCGATA	16,836,073 – 16,838,204
Hotspot 130x	GGTTTTGCCTTCTTATATATGTCC ACATC	GTCGATACCTTGTGAGATGTAG C	181,542 – 182,221
Coldspot	GCATGAAGATATGTTTAAAC	AGATGTTCTCCTATTGTGAG	16,832,332 – 16,833,680
